# Development and Evaluation of Integrated Diabetes Curricula for Teaching Gene by Environment Concepts to High School Health and Biology Students

**Published:** 2024-03-19

**Authors:** Atom Lesiak, Joan C. Griswold, Adam Moylan, Helene Starks

**Affiliations:** 1 Departments of Genome Sciences, University of Washington, Seattle WA; 2 Departments of Rockman et al Cooperative, San Francisco CA; 3 Departments of Bioethics and Humanities, University of Washington, Seattle WA

**Keywords:** STEM Education, Model Systems, Type 2 Diabetes, Interdisciplinary Curricula

## Abstract

The authors designed an integrated type 2 diabetes (T2D) curricula to model real-world complexity for high school biology and health students, highlighting interactions between genetic, biologic, environmental, and social factors, and modeling prevention and intervention activities. We evaluated the curriculum with two samples of students (888 historical comparison [no exposure] and 2,122 intervention students [received the T2D curricula]). Students completed pre-post assessments that were analyzed for knowledge gains and changes in self-efficacy to engage in healthy behaviors. Correct posttest answers in the intervention group increased by 24% versus 1% (biology) and 3% (health) of comparison students (*p* < .001); mean (sd) self-efficacy scores increased for biology [3.2 (25.2)] and health [1.5 (7.2), both *p* < .0001)]. COVID-19 prompted mandatory online teaching starting in March 2020 resulting in more health (65%) than biology students (47%, *p* < .001) doing the curriculum in virtual/hybrid classrooms, yet posttest knowledge gains were similar for these students learning in class or online (*p* = .47). Students’ “take-home” messages mentioned the importance of prevention (64%), physiological mechanisms for developing T2D (54%), and environmental factors (17%). The curricula successfully delivered cross-disciplinary content without placing undue burden on teachers to create and sustain integrated learning systems.

## INTRODUCTION

Interdisciplinary and integrated curricula in middle and high school instruction transcend disciplines and help with evidence synthesis by asking students to make connections across disciplinary concepts and examples (Applebee et al., 2007; Drake and Reid, 2020). These curricula reflect authentic ways in which fields of study interact in the real world and promote dialogue across disciplines to prepare students from a variety of populations for their next level of education (Lehrer, 2022). For example, Math and Science coordination is essential in early science, technology, engineering, and math (STEM) education to allow for mathematical support of science equations. Family and Consumer Sciences (FACS) and health classes rely on shared frameworks to reinforce how health sciences play out in personal, family, and community life.

The goal of this project was to create a cross-disciplinary model through which high school students could learn about gene-environment interactions. We chose type 2 diabetes (T2D) as a model system for several reasons; it is: 1) steadily increasing in incidence; 2) a complex, preventable condition that has real-world relevance for students and their families; 3) is influenced by gene-environment interactions that operate across multiple levels, e.g., DNA, cells, individual bodies, families, kitchens, grocery stores and public policy; and 4) is currently used in curricula in multiple disciplines to teach core concepts. In what follows, we describe the development and evaluation of an integrated type 2 diabetes (T2D) curricula to model real-world complexity for high school biology and health students, highlighting interactions between genetic, biologic, environmental, and social factors, and modeling prevention and intervention activities.

As a model system, T2D teaches about homeostasis and feedback mechanisms in biology and physiology courses; in health and FACS courses, T2D demonstrates interactions and effects between nutrition, diet, exercise, economics, and access to resources. T2D as a model represents a common, complex condition that is unevenly distributed across geographic areas, with higher prevalence amongst vulnerable and marginalized groups. The Centers for Disease Control and Prevention (CDC) predict that two in five Americans are expected to develop T2D in their lifetimes, and eight out of ten people with prediabetes today do not know they are at risk (CDC, 2023). Many increases in incidence of T2D and other multifactorial traits are driven by changes in the environment that influence gene expression. Focusing on these interactions addresses the misconception that genes are the sole determinant of one’s traits (Mills Shaw et al., 2008; Venville and Donovan, 2005).

A challenge in high school education is equipping students with foundational STEM and FACS knowledge to empower them to be educated citizens who are prepared to address complex, real-world problems that transcend one area of study. Students generally take separate courses in health, FACS, biology and genetics, and environmental science, with each topic taught in different classes over a number of years. There are few curricular links between these courses to help students understand, for example, how interactions of genes and the environment can lead to different health outcomes (McLysaght, 2022). Additionally, biology textbooks have historically relied on examples of classic single-gene traits like sickle-cell anemia or cystic fibrosis to tell the story of genetics through mendelian patterns of inheritance (Hicks et al., 2014; Kampourakis, 2021). Discussions of “the environment” in biology are often limited to the effects of temperature, pH, humidity, light cycles, and/or the presence of mutagens. Health classes may touch on a broader definition of “the environment” which may include social determinants of health such as access to community-level resources (e.g., parks, sidewalks, grocery stores, fast food restaurants, and transportation) as well as state and federal policy. Embracing this larger view of the environment in the context of genetics introduces complexity, as “unlike genetic loci, the environment is boundless” (Patel, Bhattacharya, and Butte, 2010), which may explain why multifactorial health conditions are not more thoroughly explored in biology and health classes (Griswold, 2023).

### Program History.

The seed for this interdisciplinary project took root during a week-long professional development (PD) session for our traditional audience of science teachers in rural Yakima Valley in Washington State in 2014. The goal of the workshop was to train biology teachers in our new curriculum that used the health condition of type 2 diabetes to provide context for biological concepts. During the recruitment process, health teachers expressed interest in the PD as a way of increasing science content in health classes to support students in end-of-course exams. We opened enrollment and provided PD for all interested teachers, regardless of discipline.

During the subsequent school year, program staff observed our large, 8-lesson T2D unit get split along natural lines as teachers mixed-and-matched lessons to best fit their class disciplines. Lessons teaching concepts such as homeostasis, feedback mechanisms, and the role of hormones fell to the biology classrooms. Health, FACS and physical education (PE) teachers focused on the lessons that taught nutrition, label literacy, exercise, and disease prevention. Some of the lessons and activities crossed boundaries and were taught in a variety of classes. Building on this, we began actively recruiting science, health, and FACS teachers for PD. Within a few years from the serendipitous beginning, thousands of students in our original seed school had been exposed to some portion of the T2D-related curriculum in one, two, or even three different classes.

### Curriculum Design.

**Figure 1.**
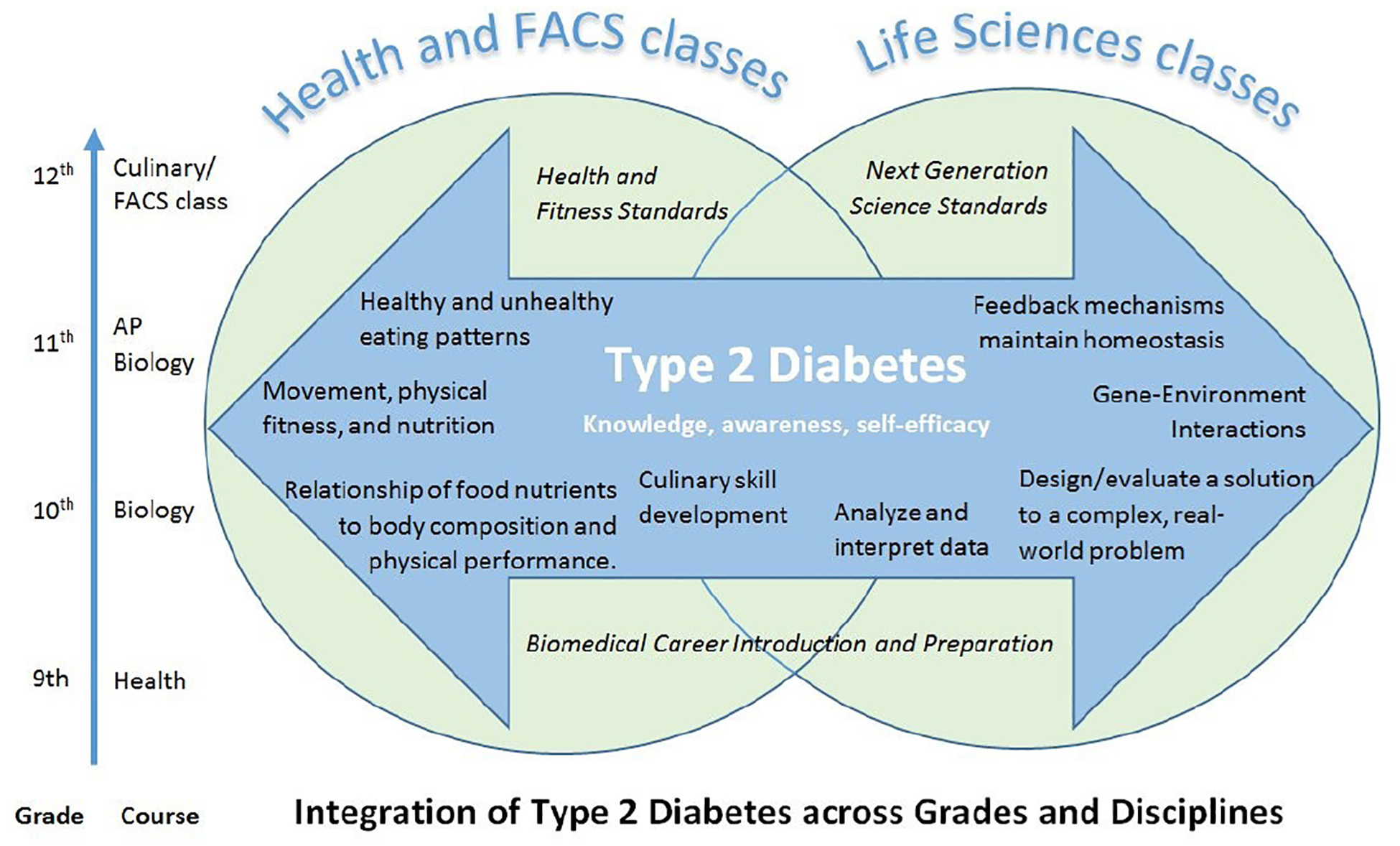
Conceptual Model of Integration of Type 2 Diabetes across Grades and Disciplines. The two curricula share core content about T2D, then have discipline-specific lessons to address standards and learning objectives. The content is relevant for students in all grades who are enrolled in biology or health classes. Extension lessons were designed for AP biology and culinary/FACS classes.

Using these experiences as a foundation, we received funding in 2017 to design and evaluate an integrated curriculum that uses T2D to deliver related content for biology and health/FACS classes without the need for teacher coordination. The curricula – independently and together – model real-world complexity and highlight interactions between biological, environmental, and social factors (Figure 1). The curricula were designed to be taught in either or both biology and health/FACS classes. Each curriculum teaches core concepts, using discipline-specific examples to promote learning and engagement (Table 1). Students who receive both curricula get similar content applied in different ways. In this way, the curricula can serve as the linkage between classrooms and disciplines without extra teacher effort for cross-disciplinary coordination.

We used the backwards design process (Wiggins and McTighe, 2005) in collaboration with teachers, program staff, and scientists during three, weeklong curriculum development workshops. We convened these stakeholders to identify eight broad “Enduring Understandings” (EUs) about T2D that summarized important ideas with lasting value beyond the classroom (Table 1). The same set of EUs provided the foundation for all learning objectives, activities, and assessments for both the Health and the Biology modules, as well as the pre- and post-tests developed for the units. Workshop teachers piloted the modules in their classes to provide feedback and edits on the lessons and materials.

Each curriculum includes five lessons (Table 2) with specific objectives, interactive learning activities, and content to address these EUs that aligned with multiple educational standards (GSEO, 2019). For biology, the lessons contributed to student competency in Next Generation Science Standards (NGSS, 2013) with a focus on scientific practices (e.g., developing and using models; analyzing and interpreting data); core ideas in life sciences (e.g., structure and processes, interactions, energy and dynamics; inheritance and variation of traits); core ideas in engineering and technology (developing possible solutions); and cross-cutting concepts (patterns; structure and function; stability and change) (NGSS, 2013). The health curriculum was designed to address each of the eight National Health Education Standards (Joint Committee on NHES, 2007), including nutrition; health promotion and disease prevention; influence of family, peers and culture on health behaviors, goal setting, communication; and decision making to enhance personal, family, and community health.

**Figure 2.**
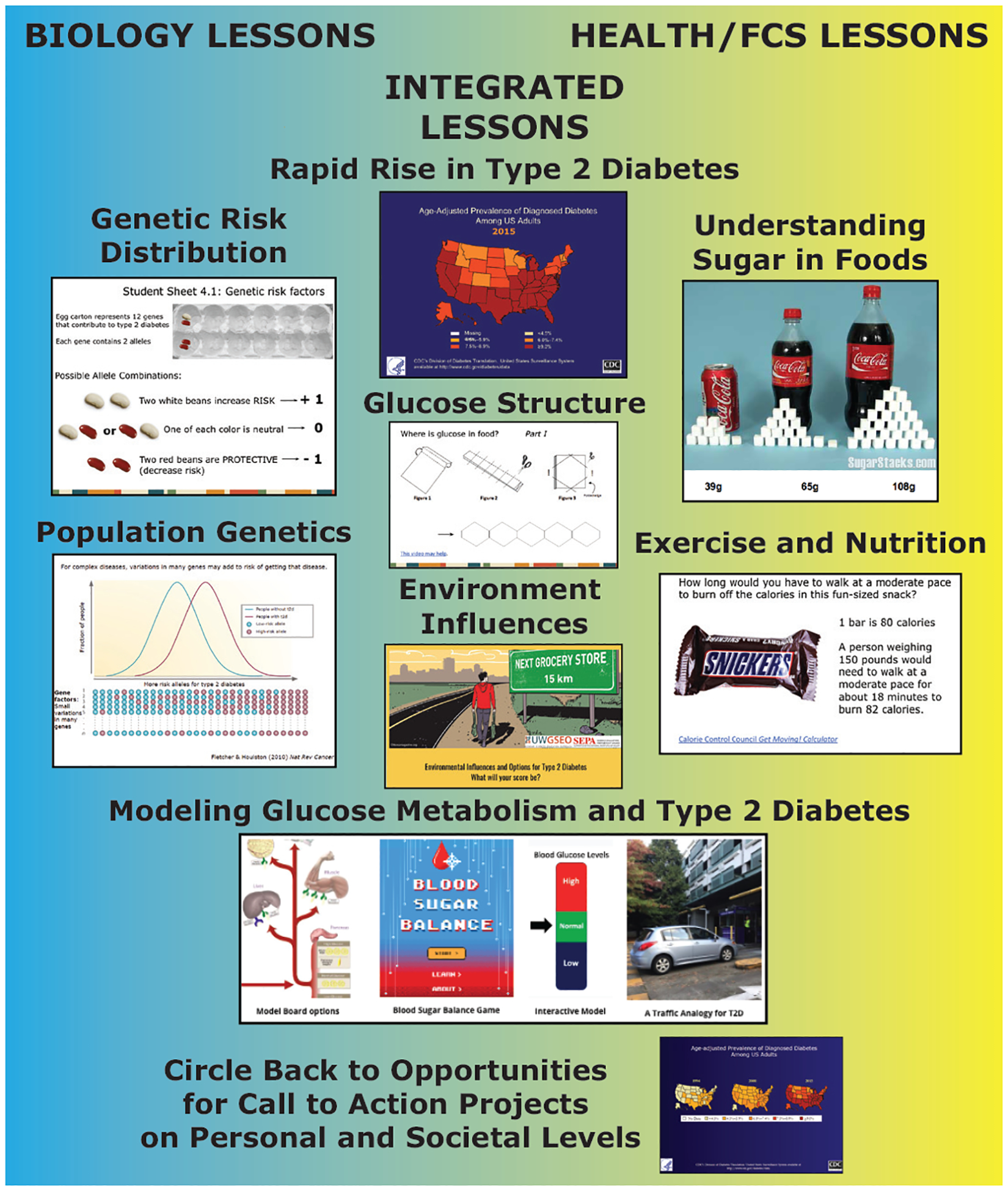
Example Lessons from the Integrated Curriculum. These snapshots are examples of class activities, some of which are shared across biology and health classes (e.g., prevalence and distribution of T2D, glucose structure, environmental influences, modeling feedback mechanisms and homeostasis). The biology specific lessons focused on the science of T2D whereas the health lessons focused on the impact of food choices on metabolism. The lessons ended with summative content to help students integrate the information as a call to action about personal and social choices.

The curriculum is inquiry-based with T2D as the driving phenomenon, and naturally integrated through the process of backwards design (Wiggins and McTighe, 2005). Each curriculum overlaps through shared activities and lesson elements that are approached from either the biological or health perspective, with additional lessons unique to each discipline (Figure 2). Lifestyle features such as food, exercise, and nutrition are emphasized in the health curriculum, while the genetic risk distribution and population genetics are exclusive to the biology curriculum. The driving question central to both curricula is the interaction of environmental influences with human metabolism to understand and explain the rapid rise in type 2 diabetes prevalence. The focus for both curricula is on prevention and helping students understand the links between biological and social mechanisms and the disease process over time and highlighting multiple points for prevention activities. For example, the curricula include several different ways to model blood glucose homeostasis and feedback mechanisms, including a physical model board, a video game, and an analogy of a parking garage with a gate attendant who regulates traffic flow. From the health perspective, these modeling activities can be approached through the lens of personal choices; from the biological perspective, through metabolic and biochemical interactions. Each of these models helps to explain glucose control mechanisms and the progression from insulin resistance to damage to the pancreas and diabetes. These models highlight and explore cross-cutting themes and systems thinking and can be found on our website: https://sites.google.com/uw.edu/t2dmodelscenarios/home.

### Curriculum Implementation.

We recruited health and biology teachers from schools across Washington state using educator listservs, presentations at statewide and national teacher conferences, and snowball sampling across multiple biology and health/FACS teacher networks. To estimate the baseline knowledge of students in these classrooms, we began with the historical comparison group in which teachers agreed to collect data from students who did not receive the T2D curriculum. Between February and September 2019, students in these classrooms completed the pre- and post-test (with 1–2 weeks between assessments) around a different unit that teachers had already planned to teach on an unrelated topic. The intervention phase ran from September 2019 through June 2021. Prior to teaching the curriculum, all intervention teachers participated in a 1–5 day workshop to learn and teach the lessons and coordinate data collection for the pre and post-tests. Teachers were invited to use the curriculum in as many classes as they could, including teaching it over multiple semesters or school years. They were encouraged to share the curriculum with other teachers in their school in both biology and health; some schools adopted the curriculum as the standard for all their biology or health teachers.

The curriculum was originally designed to be delivered face-to-face in the classroom with teachers as the primary users. In March 2020, when all Washington state public schools transitioned online due to COVID-19, we pivoted to online teacher training (Lesiak et al., 2021) and also re-designed the lessons as hyperlinked Student Roadmaps. This changed the focus to student-directed learning where the teachers used online or in-person face time to facilitate group discussions and answer questions about the lessons. The lessons were disseminated through each school’s online learning environment to students at home. This helped teachers deliver ready-made curriculum online and also created an opportunity to compare delivery mode (in class vs. online).

## METHOD

### Curriculum Evaluation.

To evaluate learning gains attributable to the T2D curricula, we used a student-level, pre-post design with a historical comparison group. The comparison group consisted of students in both biology and health classes from the participating schools that had not implemented the T2D curricula. We began instrument development by identifying the Enduring Understandings (Table 1) as the desired learning targets to be assessed. Project staff wrote content- and discipline-specific questions and augmented these with multiple choice test items about homeostasis, feedback mechanisms, and hormones culled from instruments validated for the New York High School REGENTS Exams (NYSED, 2015). Each finalized test question was linked to corresponding NGSS or health learning standards (NGSS, 2013; Joint Committee on NHES, 2007), associated with lesson objectives, rated for the level of cognitive demand, and given a point value. The instrument was reviewed and balanced throughout development with input from project staff and external evaluators using a process described by Bass and colleagues (Bass et al., 2016).

The final instrument included 45 questions to assess knowledge about the EUs taught in both curricula. It also included five self-efficacy questions that asked students to rate their confidence on a 10-point scale on being able to: 1) exercise regularly; 2) select healthy foods and drinks; 3) help their family eat a healthy diet; 4) work for community health; and 5) make changes in their own lives. Students also reported demographics including age, grade, gender, race, ethnicity, their parents’ highest level of education, and whether they are enrolled in a free or reduced-price lunch program.

The curriculum-based knowledge questions included 29 items that were relevant to both biology and health, and 16 additional questions, eight of which were specific to the biology curriculum and the other eight specific to the health curriculum (see Appendix). This meant that students answered 45 questions, but received scores based on the 37 that were relevant to their biology or health curriculum; the extra eight questions from the other curriculum served as a control for background knowledge on T2D. Students were instructed to make educated choices or select “don’t know” (vs. randomly guessing). Students were also asked to list 1–3 open-ended “take-homes” as the final question on the post test.

We computed scores as both the percent of correct answers, and as a weighted score that summed to 100 points for ease of grading and interpretation. We assigned curriculum-specific weights to the 37 questions including seven items worth one point (19%), six items worth two points (16%), 15 items worth three points (41%), and nine items worth four points (24%). In the pilot test of the instrument, student scores ranged from 30–80%, which is optimal for measuring change pre to post (Bass et al, 2016). The assessment instrument with individual item weights is included in the Appendix.

### Data Collection and Analysis.

All pre- and post-tests were administered via online links to a REDCap survey (Harris et al, 2009) Nmanaged by the University-based project staff. Teachers set aside class time and provided computer access for students to complete both the pre- and post-tests, which took an average of 18 minutes each (range 3–60 minutes). Students were advised that the tests were part of usual classroom activities and would be used for course assessment. They were given the choice to opt in or out of having their data included in study analyses. Surveys included student identifiers to allow for matching pre- and post-tests and to create student- and class-level reports with test scores reported as both percent correct and as the weighted score. These reports were shared with teachers to use as part of their course assessments, if desired. All identifiers were removed and replaced with study ID numbers after matching was complete. Teachers also completed a short exit survey to report on how they implemented the curriculum, any surprises, challenges or lessons learned about teaching it. Teachers who taught the curriculum multiple times were asked to complete a new exit survey after each round. All study procedures were reviewed and approved by the University of Washington’s Institutional Review Board.

We conducted two sets of analyses. First, we compared knowledge change scores between the historical comparison and intervention groups to attribute differences to the curricula. For the knowledge questions, we coded each response as either correct (1), incorrect (0), or don’t know (99) and computed for each student, their total scores at pre and post; “don’t know” questions were recoded to 0 for the analyses. Learning gains were measured as the difference between pre and post. We used chi-square tests to examine unadjusted differences in the proportions of student answers at pre and post, and paired t-tests to examine differences in weighted scores. Given the nested data, we also used two-level hierarchical linear modeling to estimate average treatment effects across groups, with students (level 1) nested within classrooms (level 2). Individual student scores were regressed against baseline covariates, treatment assignment, and random effects at the class level. Student level covariates were grade (9–12), age (13–18), female, race, Hispanic, and highest level of parental education. We used restricted maximum likelihood estimation to fit the linear mixed models. For each analytic sample, baseline equivalence tests were conducted prior to impact analyses using a similar multilevel model approach. The modified regression model used the pretest of the outcome as the dependent variable and included a random intercept for classroom and no other covariates. Analyses were performed using IBM SPSS Statistics (Version 28).

Second, we examined changes in health-related self-efficacy scores and the impact on knowledge scores of in-class vs. online teaching modality, using intervention group data only (these data were not collected from the comparison group). For the self-efficacy questions, we examined mean scores using the regression models described above and also recoded scores into categories of low (1–4), medium (5–7), and high (8–10) self-efficacy to graph distributions pre-post, with the results representing the data as ordinal vs. continuous measures.

### Qualitative Analysis of Student and Teacher Comments.

We conducted a directed content analysis of the students’ open-ended comments regarding course “take-homes”, using a rubric that aligned with the learning objectives for each curriculum (Hsieh and Shannon, 2005). Statements were coded as addressing one or more of these categories (*and working definitions*): prevention activities (*through lifestyle choices around diet, exercise, and nutrition*); healthy eating (*specific mention of food and healthy eating as prevention*); physiologic causes (*importance of in-range glucose homeostasis through feedback mechanisms*); generic references to causes of T2D (*“how you get it”*; *“it’s bad”*); environmental factors (*access to fast foods/sugary drinks; persistent, high levels of stress*); social/environmental justice (*contributions of income, access and education to risk factors*) or prevalence of diabetes (*it is common/serious/growing*). We also noted when students wrote a generic comment (e.g., “I learned a lot about diabetes.”), a negative one (e.g., “Nothing, it isn’t important to me to know all the scientific facts.”) or when they left it blank. We sorted the responses by codes and used the frequencies as an indicator of the major take homes from the curriculum. We also read the students’ comments within and across each code looking for the level and type of detail as a reflection of what they learned from the curriculum and activities.

In addition, we reviewed the comments from the teacher surveys to learn more about how they taught the curricula, especially with the sudden shift to virtual and/or hybrid classrooms. We asked them rate the curricula on a 5-point scale (1=Poor; 5=Excellent) and why they gave it that rating. We invited them to comment on the most important things their students learned, any surprises from students or reports of behavior changes due to the lessons, and the most important benefits of the curricula for students, teachers, and schools.

## RESULTS

### Student Characteristics.

About two-thirds of eligible students in both the comparison and intervention groups provided complete, matched pre-post data. The comparison group included 888 out of a total possible 1,333 students (67%) from 12 schools, and the intervention group included 1,964 out of 3,123 students (63%) from 19 schools. Table 3 summarizes the characteristics of the students with matched data and their classrooms. Students were comparable between the groups on all characteristics except for grade and highest level of parental education (more 9th graders and more college-educated parents in the intervention group). Neither of these covariates were significant in the regression analyses. There were differences in the proportion of online students between biology (47%) and health (65%) classes (*p* < .001) because the health classes were due to teach the T2D intervention unit in the Spring 2020 semester, which coincided with the start of the pandemic.

### Knowledge Gains.

**Figure 3.**
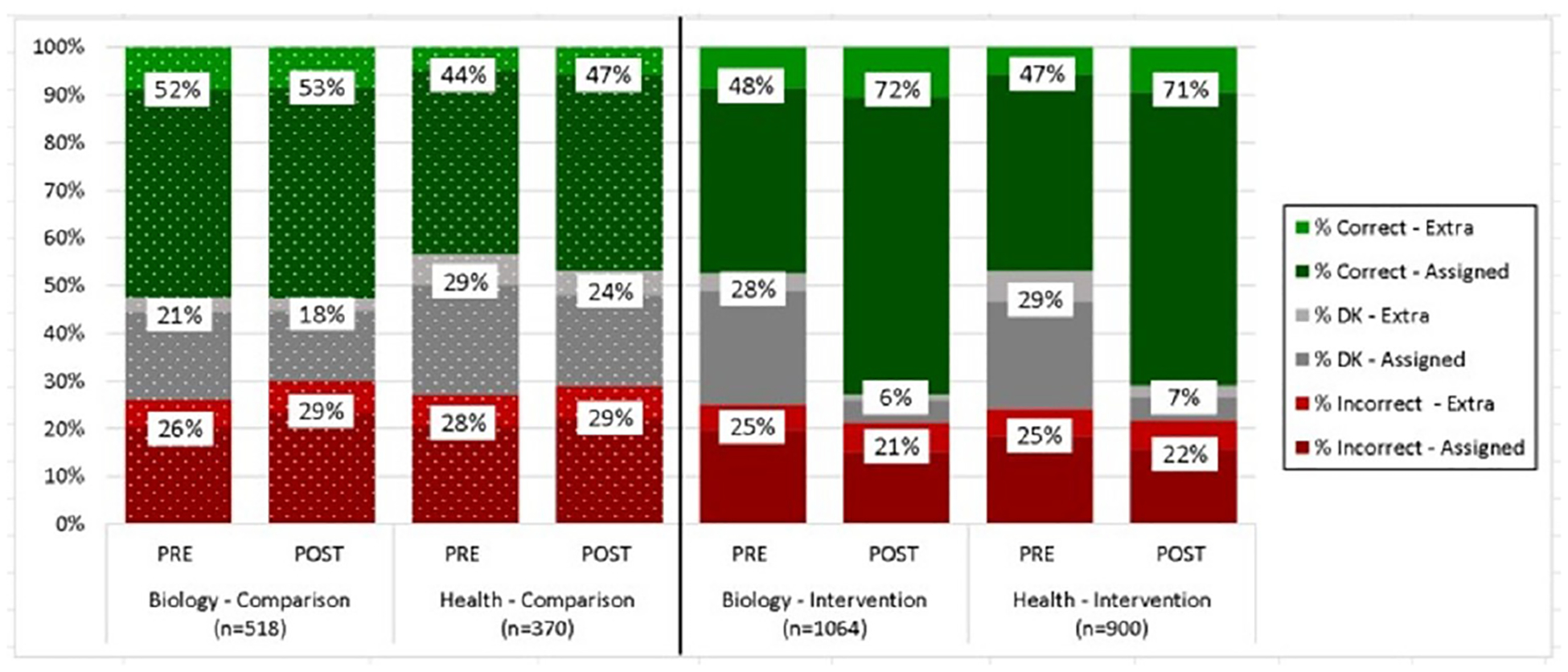
Pre-Post Scores Biology & Health Students by Comparison vs. Intervention Group. The distribution of scores shows comparable baseline scores between the groups, and significant increases in the percent of correct answers at post-test for the intervention students in both biology and health classes (*p* < .001), largely by converting “don’t know” (DK) responses to correct answers. There is also an increase in correct answers for the interdisciplinary content as measured by the 8 extra questions that were not counted in the overall score.

Figure 3 reports the unadjusted proportions of knowledge test responses and shows that, as expected, there were no differences between the pre-post scores of the comparison group, and that pre-test scores for the intervention group are similar to the comparison group. Students in intervention biology and health classes showed a 24% increase (*p* < .001) in the proportion of correct post-test answers for both the assigned (discipline-specific) and extra questions (specific to the other discipline and not covered by the assigned curriculum). These findings were confirmed in the regression analyses in both biology (*B* =.25, *p* < .001, 95% CI [.218, .276]) and health (*B* =.36, *p* < .001, 95% CI [.308, .417]) classes.

Similarly, using the change in weighted scores as the measure, the average treatment effect of the intervention was 38 points for biology students and 34 points for health students. In our assessment of teaching modality, we found no differences in the proportion of correct post-test knowledge responses when taught in class or online (biology: 76% vs. 75%, *p*=0.47; health: 71% vs 76%, *p* = .09).

**Figure 4.**
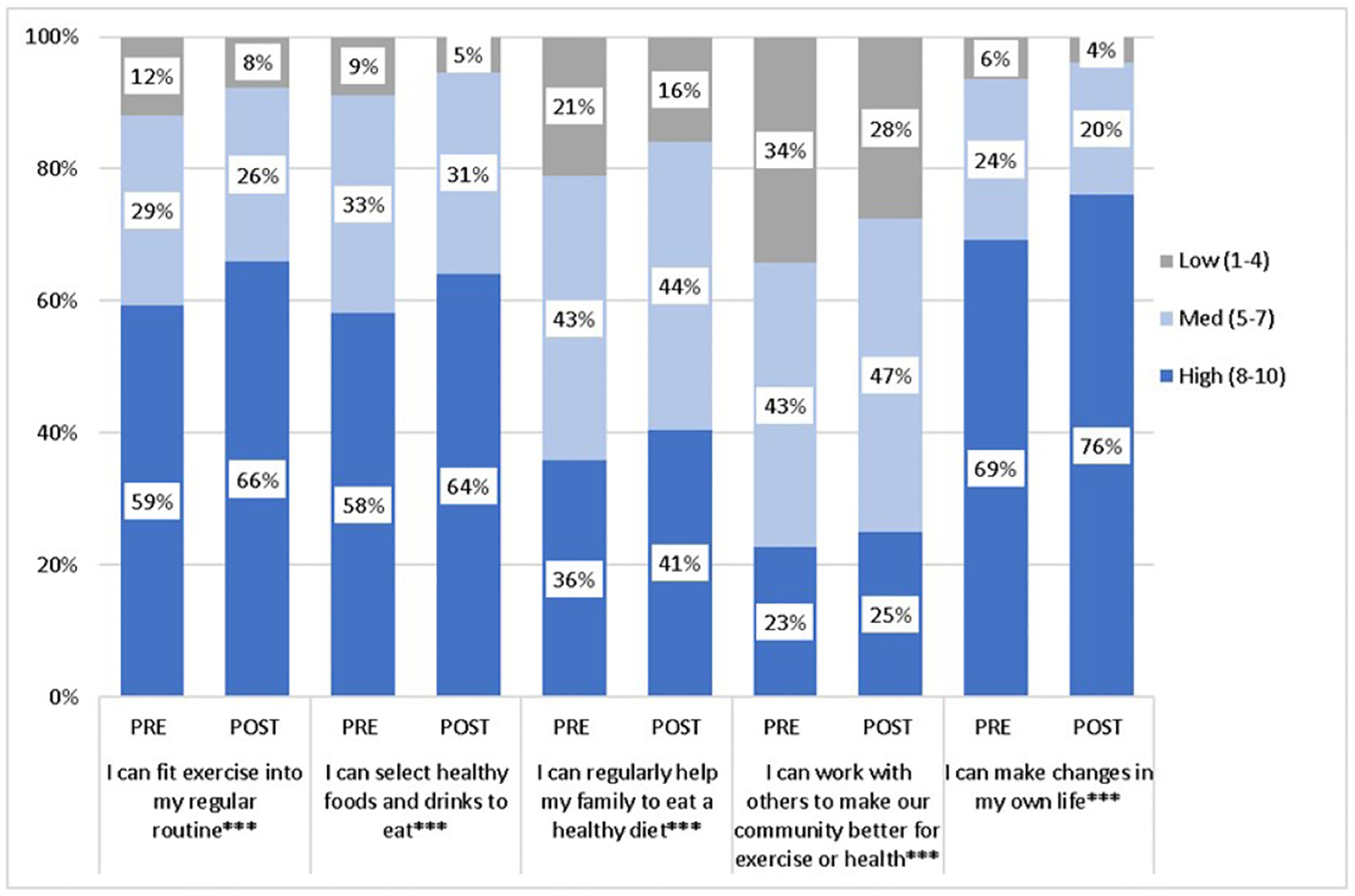
Pre-Post Self Efficacy Scores for Intervention Biology & Health Students. Intervention students in both classes reported higher self-efficacy scores for all 5 measures on the post-test (*** *p*< 0.001) with much of the shift moving from low to medium or high self-efficacy, especially for the items measuring personal behavior change.

Figure 4 reports the change in self-efficacy scores for each of the five health behaviors. We report scores for all intervention students in both biology and health classes as there were no differences between students in those classes. In both the unadjusted and adjusted analyses, self-efficacy scores significantly increased by an average (*SD*) of 2.4 (19.3) points (pre: 35.3 (9.2); post: 37.7 (19.6); *p* < .0001).

### Student “Take-Homes.”

In response to the question “What are 1–3 important or useful things you learned in this unit?” 1,899 intervention group students (89%) wrote at least one comment (range 1–6, *M* = 2.2, *SD* = 1.3). The majority (*n* = 1,348, 64%) wrote that diabetes is preventable (“I learned what I can do to lower my risk of T2D) and among these, 904 (43%) specifically cited the importance of healthy eating (“Exercise and eat healthy to try to prevent T2D”). Taken together, 54% of students made either specific references to the physiological mechanisms (“T2D is caused from constant high blood glucose levels and insulin resistance”; *n* = 271, 27%) or generic reports about how T2D works (“What T2D is and how it’s caused”; *n*=576, 27%). The environment was mentioned by 357 students (17%) (“Obesity is growing rapidly in the US. and that environmental factors [are] a huge factor on this.”). About 10% (*n* = 212) of the students mentioned social factors (“T2D tends to be more common among people of different races because their countries advertise sugary drinks more”). Another 10% (*n* = 220) commented on the rise in prevalence (“By 2050, 1 in every 3 people will have T2D if we continue with the trends we have now”). These comments reflect that students were responding to the Enduring Understandings that integrated health and biology concepts for both sets of lessons.

### Teacher Reflections.

Both biology and health teachers rated the overall curriculum as 4.1/4.2 out of 5. Noted strengths were the variety of activities, focus on active learning and inquiry, and the application of models and simulation to help the students translate the concepts to their own lives. A common sentiment was expressed by this biology teacher:

I think it is a good example of a phenomenon-based unit, that kept students engaged and interested in answering the driving question for the entire unit. There were also unique, hands-on activities (model boards, bean activity) that taught fundamental concepts in innovative and effective ways.

Teachers appreciated the curriculum’s ability “to make science relatable” and “spark conversation about risk factors and personal health habits that can lead to a multitude of teachable moments.” A common student surprise was the awareness of “how much sugar the average American gets versus what they ‘should’ be getting. They were able to look at some of their favorite drink orders (*Starbucks, Dutch Bros*.) and see exactly how much sugar they are ingesting.” The activities and discussions led students to reflect on their own consumption habits: “We had lots of great conversations about community-based environmental risks and how/why the number of fast-food restaurants would increase a person’s risk. Students were both surprised and concerned by this.” Teachers also thought that the integrated curricula allowed students to “[see] the relatedness between health and biology” and “the fact that it links so many complex biological systems with social ones is really amazing. That the curriculum is seen by every student as personal makes a huge difference to their learning.”

The quality of the lessons and the ready move to the online format was a “life saver” for teachers as they knew the content would engage students working on their own. One health teacher wrote:

In a remote learning setting, students were able to participate in a variety of activities that require and demand critical and creative solutions skills. With the diversity of students’ backgrounds in my class, it was able to meet students where they are at as well as challenge them. For my students who are “fine” with remote learning, the curriculum was not hard to understand or follow. For some of my students, it was essential that I go over each step with them even with my modifications on the roadmaps to ease the processing of information and task. I think the lessons are just as effective for those that fully participate in the lesson and activities as those that go through the lessons in person.

Several teachers commented on how the lessons allowed them to use their “air time” with students to focus on clarifying concepts and helping those who were “struggling” by teaching them how to use the roadmaps. “It was such a load off to have these lessons fully ready to go and ready for students (road map with link) because it is very stressful right now and way too much computer time for teachers to take all their curriculum online. This was SO helpful.”

## DISCUSSION

Multifactorial conditions such as T2D are complex by nature and can have an important role in the biology classroom. Strong cases have been made for inverting the genetics curriculum to begin with teaching complex conditions before teaching single-gene traits to reduce genetic predeterminism and better understand development and environmental risk factors (Dougherty, 2009; Kampourakis, 2021). Teaching multifactorial conditions in a social context also provides a target for solutions in ways that a focus on single-gene traits and individualized genetics cannot (Griswold, 2023).

The results from this project demonstrate that our integrated curricula were successful in teaching both health and biology students about the biological and environmental interactions that together contribute to the development (and prevention) of T2D. We identified three key contributing factors for developing and implementing successful integrated curricula.

1. **Identify broad learning targets that can be met through multiple disciplines.** This helps build a structure that reaches across educational silos that are often separated by grade and discipline. We identified eight Enduring Understandings—important ideas with lasting value beyond the classroom—that anchored all lessons, activities, and assessments for both units (Wiggins and McTighe, 2005). These understandings encompass ideas both micro (glucose is released through the digestion of carbohydrates) and macro (T2D occurs frequently in our communities). When all curricular resources point towards the same learning outcomes regardless of the class, those learning outcomes become automatically prioritized. In this way, students benefit from multiple exposures to the same important ideas through different classes. In building a foundation from the same Enduring Understandings for our interdisciplinary units, we were also able to measure learning outcomes across disciplines.
2. **Build in coordination and structure** through the curriculum without placing undue burden on individual teachers for creating and sustaining integrated systems. A pragmatic challenge in interdisciplinary education of the need for coordination between teachers across classrooms. To address this, we built upon the same Enduring Understandings, embedded a diabetes-related anchoring phenomenon into each unit, and incorporated the BSCS 5E learning cycle structure (Bybee, 1997) to create two stand-alone curricula that each meet discipline-specific educational standards yet complement each other in structure and learning outcomes. The scaffolding of the parallel curricula allows it to integrate itself into classes, thereby relieving teachers of the need to meet with their peers to create connections between courses. Students who experience parallel themes across classes are more likely to recognize the interconnections than the teachers who are familiar with only their portion of instruction.
3. **Choose a topic that is rich in complexity**. Integrated curricula thrive when the topic lends itself to student questioning in many interrelated areas. The rapid rise in T2D in the United States was chosen as an authentic meaningful problem in need of cross-disciplinary solutions to explore questions related to genetics and other biological concepts, influences on individual choices, access to resources, product marketing, public policy, socio-economic status, strategies for prevention, and more. Many biology and health textbooks use the actions of insulin and glucagon to illustrate homeostasis and feedback mechanisms but stop short of examining important environmental and social factors that contribute to type 2 diabetes diagnoses. A complex system is made of nodes at both micro and macro levels; interactions and the model system allow for measurement and observation at each level. We sought to add structure to complexity by identifying Enduring Understandings that focus on different levels—micro to macro--of T2D. The solid framework that contextualized details within the big picture, allowed the curricular units to move between levels in an integrated way. Additionally, teachers and students both commented on the engaging nature of socially contextualized lessons in which there will be no answer in the back of the book.

A complex topic is an asset when developing curricular extensions. Once the foundational units used in the formal research study were completed, we used the same structures to develop additional lessons with ties to type 2 diabetes. These include a two-lesson series on sugar, the mechanism of taste, and cell communication; how food choices and the environment affect the gut microbiome; how societal factors can drive health disparities; and how decisions are made about access to resources such as insulin (all available at https://sites.google.com/uw.edu/gseo-online-lessons/home and https://gsoutreach.gs.washington.edu/). Additional expansion units could be developed focusing on interventions and treatments for T2D, such as how GLP-1 agonists (e.g., Ozempic) work to treat T2D.

### Moving Online.

The inherent structure of the modules was key to the successful migration from in class to online learning during COVID-19 restrictions. We created hyperlinked Student Road Maps following the same 5E model as the original teacher-centered units to facilitate direct student access to lesson activities and concepts. Online videos supported student activities, teacher demonstrations, and substituted for direct instruction. Icons were inserted in the road maps in places rich for discussion so that valuable online face-to-face time could be focused on discussion rather than direct instruction. Multiple revisions of our popular homeostasis model led to the development of the online Blood Sugar Balance game (https://sites.google.com/uw.edu/bloodsugarbalance/home for use in both biology and health classes (Lesiak and Griswold, 2023). The primary hurdle in moving online was finding, using, and training teachers in the platform for curriculum dissemination.

It is important to note that study was not designed to measure the differences between in-person and online administration of the curriculum. The minimal differences in student knowledge gains online vs. in-person in both curricula is a testament to the successful conversion from in-person to online. We credit the power of engaging students in an authentic real-world problem coupled with the robust structures we built into the curriculum in facilitating this transition. Regardless, the transition online was not without challenges. Teacher comments highlighted differences between students who easily navigated self-directed learning compared to others who really struggled and needed substantial teachers support. An additional concern in the online space was students having access to online resources and information to artificially enhance their knowledge gains on assessments. We cannot rule out the possibility that access to online resources might have masked an overall decrease in knowledge gains while using the online curriculum, and support continued research into the characteristics of online and self-directed education.

### Conclusion.

Developing an integrated curriculum of this nature requires an upfront investment of work during initial development, that then pays dividends in myriad ways. This study provides further evidence supporting both this curricular design approach, and of a successfully integrated curriculum built around the phenomenon of T2D. Educators and education researchers can use this work as a model for future integrated curriculum development around other topics that can or might cross disciplines.

## Supplementary Material

Appendix

## Figures and Tables

**Table 1.** Enduring Understandings for the Biology and Health T2D Curricula.

Most traits are determined by a combination of genetic and environmental factors, including complex diseases like type 2 diabetes. Type 2 diabetes is a growing concern and occurs frequently in our communities. Type 2 diabetes is a complex condition that is heavily influenced by environmental factors such as access to resources, personal choice, product marketing, public policy, socio-economic status, and stress. Type 2 diabetes is caused by the effects of high blood glucose levels over time. Glucose, the major energy source for all human cells, is released primarily through digestion of carbohydrates. Food choices impact blood glucose levels. Type 2 diabetes is a serious condition with negative health consequences if left untreated. Type 2 diabetes can be prevented: factors contributing to a person’s risk include good nutrition and exercise. Students can make a meaningful contribution to the prevention of type 2 diabetes.

**Table 2.** Topics addressed in the biology and health curricula.

Lesson	Biology Curriculum^1^	Health Curriculum^2^
1	**Asking questions about diabetes** Students explore a CDC slide presentation that shows the rapid increase in diagnosed cases of T2D in the past 20 years to ask questions about how diabetes diagnoses are impacted by age, educational level, geography and other factors.	**Genes and the environment** Silent Chalk Talk starts a conversation about beliefs about diabetes, followed by exploring the CDC slides to examine trends in diabetes and obesity.
2	**Homeostasis—glucose in balance** Students are introduced to glucose homeostasis through a model that shows how organs and systems interact through feedback mechanisms to maintain balance. As an extension, students use yeast as an indicator for cellular respiration.	**Our environment: Access and choice** Students learn how T2D is influenced by our environments and assess their own environmental risk factors for T2D. Students learn how the change in environment for one population has impacted their health over time.
3	**Modeling type 2 diabetes** Students collect evidence for the causes of T2D by using the homeostasis model board to figure out how blood glucose homeostasis is affected by diet, exercise, insulin resistance, and pancreatic function.	**Sugar: From fuel to toxin** Students learn how T2D is influenced by our environments and assess their own environmental risk factors for T2D. Students learn how the change in environment for one population has impacted their health over time.
4	**Genes and environmental risk factors** Students learn about environmental, genetic and social factors that influence T2D by simulating how high risk and low risk gene variants may be distributed through a population and looking for patterns in their own environments and eating habits.	**What are we eating?** Students examine food and drink labels to figure out how different types of food impact blood glucose levels. They calculate the percentage of proteins, fats, and carbohydrates contained in different foods and drinks, and visually illustrate liquid sugars in a beverage.
5	**Evaluating solutions** Students use evidence gathered throughout the unit to generate an argument in support the best treatments and preventative measures that address this complex condition.	**An ounce of prevention** Students learn ways in which exercise can aid in treating and preventing T2D and determine physical activity requirements for balancing calories consumed and burned.

1 https://sites.google.com/uw.edu/gemnet-bio-t2d-curriculum/home

2 https://sites.google.com/uw.edu/gemnet-health-t2d-curriculum/home

**Table 3.** Student demographics by comparison vs. intervention group.

N	Comparison (No curriculum)						Intervention					
	Total	%	Biology	%	Health	%	Total	%	Biology	%	Health	%
	888		518	58%	370	42%	1964		1064	54%	900	46%
*Grade in School* ***												
9th	524	59%	257	29%	267	30%	1365	70%	716	36%	649	33%
10th	278	31%	220	25%	58	7%	424	22%	310	16%	114	6%
11th	61	7%	36	4%	25	3%	77	4%	16	1%	61	3%
12th	25	3%	5	1%	20	2%	98	5%	22	1%	76	4%
*Gender*												
Male	407	46%	231	26%	176	20%	890	45%	461	23%	429	22%
Female	462	52%	273	31%	189	21%	1023	52%	577	29%	446	23%
Transgender/ Non-binary	15	2%	11	1.2%	4	0.8%	49	2%	26	1%	23	1%
Not answered	4	0%	3	0%	1	0%	6	0%	2	0%	4	0%
*Race*												
White	571	64%	355	40%	216	24%	1197	61%	663	34%	534	60%
Black/African American	50	6%	15	2%	35	4%	106	5%	41	2%	65	7%
Asian	42	5%	23	3%	19	2%	140	7%	74	4%	66	7%
American Indian/Alaskan Native	39	4%	25	3%	14	2%	70	4%	44	2%	26	3%
More than one race	68	8%	46	5%	22	2%	176	9%	85	4%	91	10%
Other	114	13%	51	6%	63	7%	269	14%	155	8%	114	6%
Not answered	4	0%	3	0%	1	0%	6	0%	2	0%	4	0%
*Hispanic/LatinX*												
No	724	82%	433	49%	291	33%	1616	82%	876	45%	740	83%
Yes	157	18%	80	9%	77	9%	338	17%	185	9%	153	17%
Not answered	7	1%	5	1%	2	0%	10	1%	3	0%	7	1%
*Highest level of parents’ education* ***												
Less than high school	113	13%	64	7%	49	6%	189	10%	112	6%	77	9%
High school diploma/GED	120	14%	80	9%	40	5%	239	12%	126	6%	113	13%
Community college/ trade school	197	22%	122	14%	75	8%	411	21%	209	11%	202	23%
College/university grad	276	31%	165	19%	111	13%	692	35%	391	20%	301	34%
Graduate degree	138	16%	69	8%	69	8%	371	19%	200	10%	171	19%
Don’t know	44	5%	18	2%	26	3%	62	3%	26	1%	36	4%
*Teachers*	21		12	57%	9	43%	37		22	60%	15	40%
*Classrooms*	58		37	64%	21	36%	137		73	53%	64	47%
*Modality* ***							1964		1064		900	
In-class	888		518	58%	370	42%	1048	53%	749	70%	315	34%
Online	0						916	47%	299	29%	601	65%

*** p<0.001

## ABBREVIATIONS

CDC: Centers for Disease Control and Prevention
DK: Don’t Know
EU: Enduring Understandings
FACS: Family and Consumer Sciences
GEMNet: Genes, the Environment, and Me: A Health and STEM Network
GSEO: Genome Sciences Education Outreach
PD: Professional Development
PE: Physical Education
SEPA: Science Education Partnership Award
STEM: Science, Technology, Engineering, and Math
T2D: Type 2 Diabetes
