# Supplementary material for: Development and Evaluation of Integrated Diabetes Curricula for Teaching Gene by Environment Concepts to High School Health and Biology Students
Source: J STEM Outreach. Author manuscript; Available in PMC 2024 Mar 28. (PMC10976998)
Supplement: Appendix [file NIHMS1978838-supplement-Appendix.pdf]

Integrated T2D Curricula for Health & Biology Classes – Lesiak, et al.
Appendix. Assessment Questions

APPENDIX A. Assessment questions with weights for biology and health students

QUESTION [Correct answer]	Weights	Biology	Health
1. Which of the following statements about type 2 diabetes are true?		Points per Q	
1a. Being younger than 30 is a risk factor [FALSE]		3	3
1b. It is more common in certain locations in the United States [TRUE]		3	3
1c. It is mostly caused by defective genes [FALSE]		3	3
1d. It is associated with being obese [TRUE]		1	2
2. The following are some reasons why type 2 diabetes an important public health focus in the United States. Which of these statements is true?			
2a. It is especially common among wealthy people. [FALSE]	(Health only)	0	1
2b. Diabetes is growing rapidly in the United States [TRUE]		1	1
2c. Type 2 diabetes is the most common form of diabetes [TRUE]		1	2
2d. Currently, there are no ways to prevent type 2 diabetes. [FALSE]		3	3
3. Damage to the body from type 2 diabetes is due to long periods of time with: [High blood glucose levels]		2	2
4. A solution to the problem of type 2 diabetes that impacts the most people will likely focus on: [Environmental factors]	(Biology only)	4	0
5. Which is a healthier snack for someone sitting and doing homework? [The apple because it contains a lot of fiber]		0	3
6. Fill in the blank for the process below. oxygen + __________] ==> carbon dioxide + water + energy [Glucose	(Biology only)	2	0
7. If a food has 10 grams of fat and 100 calories per serving, what is the percentage of calories from fat in one serving? [90%]	(Health only)	0	4
8. What determines a person's height? [Both genetic and environmental factors]	" "	0	4
9. Approximately how long would a 130-pound person need to walk at a moderate pace to burn off an 80-calorie Fun Size Snickers bar? [25 minutes]	" "	0	4
10. Which of the following are true statements about how exercise helps prevent type 2 diabetes?			
10a. Muscles use blood glucose for energy during exercise [TRUE]		2	3
10b. With exercise, muscle cells are better able to take up glucose even if insulin is not present [TRUE]		3	3
10c Muscles convert more blood glucose to glycogen during exercise than during rest [FALSE]	(Biology only)	4	0
10d. Burning calories through exercise helps to maintain or decrease weight [TRUE]		1	1
11. What is the advantage of releasing insulin after eating? [It helps the body use food properly by letting glucose enter the cells]		4	4
12. What is substance A? [Insulin]		3	3
13. What is substance B? [Glucagon]	(Biology only)	4	0
14. The interaction of substances A and B is an example of: [A feedback mechanism]	" "	4	0
15. Which treatment has the best effect on preventing the development of type 2 diabetes? [Lifestyle changes]	" "	2	0
16. Which of the following statements is true? People in: [Genetic risk group 4 should make lifestyle changes to prevent type 2 diabetes as Metformin does not help them much]	" "	3	0
17. The graph represents: [The maintenance of homeostasis]	" "	4	0
18. How might these results be different if this person was not able to make enough insulin? [The maximum level of blood glucose would be higher]		4	4
19. The substance that is responsible for the decrease in blood glucose concentration is released by: [The Pancreas]		3	4

1

QUESTION [Correct answer]	Weights	Biology	Health
20. How does exercise impact a person's risk for type 2 diabetes? [Exercise decreases the risk by making muscles better able to take up glucose from the bloodstream]		2	2
21. Which of the following decreases the risk of developing type 2 diabetes? [Walking 1/2 mile or more to school or work each day]		2	3
22. Which of the following statements are true?			
22a. Canned kidney beans and Coca-Cola have the same effect on blood glucose levels [FALSE]	(Health only)	0	1
22b. Coca-Cola causes the largest spike in blood glucose levels [TRUE]		1	1
22c. Eating salmon has no effect on the Blood Glucose Index [FALSE]	(Health only)	0	2
22d. The glucose from whole oats is released more slowly than the glucose from Coca Cola [TRUE]	" "	0	1
23. How do the following neighborhood features affect the risk of developing type 2 diabetes?			
23a. Convenience stores such as 7-Eleven [Increases risk]	" "	0	2
23b. Fast food restaurants [Increases risk]		1	1
24. What class of molecules digest carbohydrates? [Enzymes]		1	1
25. An example of a polysaccharide (complex carbohydrate) is: [Fiber]		3	3
26. An example of a monosaccharide (simple carbohydrate) is: [Glucose]		4	4
27. Based on this graph, what claim can you make for the relationship between education level and incidence of diabetes? [The higher the education level, the lower the incidence of diabetes]		4	4
28. Which of the following might explain these patterns? [People with lower education levels tend to have lower paying jobs and less access to healthy foods they can afford]		3	3
29. Which of the following pairs contribute to type 2 diabetes? [Insulin resistance and damage to the pancreas]		3	3
30. Line 1 represents: [A person with diabetes]		3	4
31. Line 2 represents: [A person with pre-diabetes]		3	3
32. Line 3 represents: [A person with normal glucose regulation]		3	3

2
